# Distinct Effects of Social Stress on Working Memory in Obsessive-Compulsive Disorder

**DOI:** 10.1007/s12264-020-00579-3

**Published:** 2020-10-01

**Authors:** Qianqian Li, Jun Yan, Jinmin Liao, Xiao Zhang, Lijun Liu, Xiaoyu Fu, Hao Yang Tan, Dai Zhang, Hao Yan

**Affiliations:** 1 Peking University Sixth Hospital, Beijing, 100191 China; 2 Peking University Institute of Mental Health, National Health Commission Key Laboratory of Mental Health (Peking University), National Clinical Research Center for Mental Disorders (Peking University Sixth Hospital), Beijing, 100191 China; 3 Tsinghua University-Peking University Joint Center for Life Sciences, Beijing, 100871 China; 4 PKU-IDG/McGovern Institute for Brain Research, Peking University, Beijing, 100871 China; 5 Lieber Institute for Brain Development, Baltimore, MD 21205 USA; 6 Department of Psychiatry and Behavioral Sciences, Johns Hopkins University School of Medicine, Baltimore, MD 21205 USA

**Keywords:** Working memory, Acute stress, Obsessive-compulsive disorder, Functional magnetic resonance imaging

## Abstract

Stress might exaggerate the compulsion and impair the working memory of patients with obsessive-compulsive disorder (OCD). This study evaluated the effect of stress on the cognitive neural processing of working memory in OCD and its clinical significance using a “number calculation working memory” task. Thirty-eight patients and 55 gender- and education-matched healthy controls were examined. Stress impaired the performance of the manipulation task in patients. Healthy controls showed less engagement of the medial prefrontal cortex and striatum during the task under stress *versus* less stress, which was absent in the patients with OCD. The diagnosis × stress interaction effect was significant in the right fusiform, supplementary motor area, precentral cortex and caudate. The failure of suppression of the medial prefrontal cortex and striatum and stress-related hyperactivation in the right fusiform, supplementary motor area, precentral cortex, and caudate might be an OCD-related psychopathological and neural response to stress.

**Electronic supplementary material:**

The online version of this article (10.1007/s12264-020-00579-3) contains supplementary material, which is available to authorized users.

## Introduction

Obsessive-compulsive disorder (OCD) is a chronic and disabling mental disease [1, 2] characterized by the presence of obsessions (repeated thoughts) or compulsions (repeated behaviors to neutralize anxiety), or both [3]. The World Health Organization ranks it as one of the ten most disabling conditions by lost income and reduced quality of life [4].

Stress is one of the main risk factors for the formation of the disorder [5], and previous studies have indicated that OCD patients have an impaired stress response [6]. The neuropsychological theories of OCD suggest that it might be a disorder of imbalance between goal-directed behavior and maladaptive habits [7]. The excessive habit formation or habit dysregulation might result in the compulsions in OCD, which can be exaggerated by stress [8]. Moreover, stress impairs working memory (WM) and cognitive flexibility; this has been confirmed by meta-analysis of the main neuropsychological assessments in OCD patients [9]. Meanwhile, WM has been found to shape and moderate the balance between the goal-directed and habitual systems as one of the core cognitive abilities [10, 11].

Accumulating evidence from neuroimaging studies has suggested a certain extent of spatial overlap between the neural mechanism of OCD, the stress effect, and the WM process [12]. The well-known neuropathological model suggests that the function of the cortical-striatal-thalamo-cortical (CSTC) loop is impaired in OCD [13–15]. Banca *et al*. found that, during effective symptom provocation, OCD patients show reduced activation in the ventromedial prefrontal cortex (vmPFC) and caudate nucleus, and increased activation in regions of the pre-supplementary motor area (pre-SMA) and putamen, which have also been implicated in goal-directed behavioral control and habit learning, respectively [16]. A visuospatial WM study suggested an inefficient fronto-parietal executive network and increased connectivity between frontal regions and the amygdala in OCD [17]. Moreover, meta-analysis of the brain responses to the stress of trauma revealed hyperactivation in the amygdala, insula, and precuneus cortex and hypoactivation in the anterior cingulate cortices and vmPFC [18, 19]. Some studies have indicated that stress leads to changes in the neuroendocrine and limbic brain, resulting in mental diseases such as depression and post-traumatic stress disorder [20, 21]. However, how stress influences the neural processing related to the cognitive deficits of WM in OCD and the clinical correlations remain unknown.

Therefore, in the current study, a new event-related “number calculation working memory” task which integrated interpersonally competitive stress into the WM process was used to investigate the stress effect on WM in OCD. This newly designed task differs from the Montreal Imaging Stress Task (MIST) [22] which consists of mental arithmetic under social evaluating stress but not WM. Based on our previous work in a large sample of healthy adult Han Chinese individuals [23], the task-related activity included the prefrontal-parietal system and stress-related suppression of the medial prefrontal cortex (mPFC) and striatum. Therefore, in view of the neuropathological theory of OCD, we hypothesized that the regions of the CSTC circuit, frontal-parietal executive, and stress-related networks might respond differentially to the WM task under stress in patients with OCD *versus* healthy controls.

## Materials and Methods

### Ethics Statement

The Ethics Committee of the Peking University Sixth Hospital approved the study. All participants were given detailed information regarding the purpose and procedures of the study. Only patients who could consent were invited to participate. All the participants enrolled gave written consent.

### Participants

In total, 125 individuals participated in the study: 58 patients with OCD and 67 healthy controls (HCs). The patients were recruited from either the inpatient or outpatient department of Peking University Sixth Hospital. Patients all met the Diagnostic and Statistical Manual of Mental Disorders, 4th Edition, Text Revision (DSM-IV-TR) diagnostic criteria of OCD, without other comorbidities in the DSM-IV-TR Axis I Disorders (including depression), and were evaluated by two psychiatrists using the Structured Clinical Interview for DSM-IV-TR Axis I Disorders, Patient Edition (SCID-I/P). The exclusion criteria were any comorbidity of other SCID-I diagnoses, electroconvulsive therapy within six months, or a history of severe medical illness. All HCs were recruited from the local community by advertisement and assessed by psychiatrists using the SCID-I, Non-Patient Edition to exclude any mental disorder. Participants were excluded if they had: a history of neurological disease, a history of > 5 min loss of consciousness, or magnetic resonance imaging (MRI) contraindications.

The Yale-Brown Obsessive Compulsive Symptom Checklist Scale and Severity Scale (YBOCS-CS/YBOCS-SS) [24], Hamilton Anxiety Scale (HAMA) and Hamilton Depression Scale (HAMD), and four factors were used to assess the symptom severity and dimensions in patients. In the 38 patients included in the final analysis, 24 were taking one or more antidepressants and 14 were drug-naïve. All medication doses were transformed into Imipramine equivalents (mg/day) [25] (see Tables 1 and S1 for details).

**Table 1:** Demographics and clinical data of the obsessive-compulsive disorder patients (OCD) and healthy controls (HC).

Variables	OCD (*n* = 38)*	HC (*n* = 55)*	*t*/*χ*^*2*^	*P* value
Gender (F/M)	14/24	30/25	2.83	0.09**
Age (years)	27.1 ± 6.1	23.6 ± 2.9	3.25	0.02†
Edu (years)	15.7 ± 2.7	16.4 ± 1.6	− 1.32	0.19†
Imipramine equivalents (mg/day)	232.6 ± 128.8			
Course (months)	77.0 ± 47.1			
YBOSC-TS	21.9 ± 13.8			
YBOSS-TS	23.0 ± 7.8			
HAMAS	12.0 ± 7.6			
HAMDS	8.9 ± 6.2			

*Unless otherwise indicated, data are the mean ± SD; **Pearson *χ*^2^ (categorical data); †independent-sample *t*-test (parametric data); YBOSC-TS, Yale-Brown Symptom Checklist total score; YBOSS-TS, Yale-Brown severity scale total score; HAMAS, Hamilton Anxiety Scale total score; HAMDS, Hamilton Depression Scale total score.

### Working Memory Paradigm

**Fig. 1:**
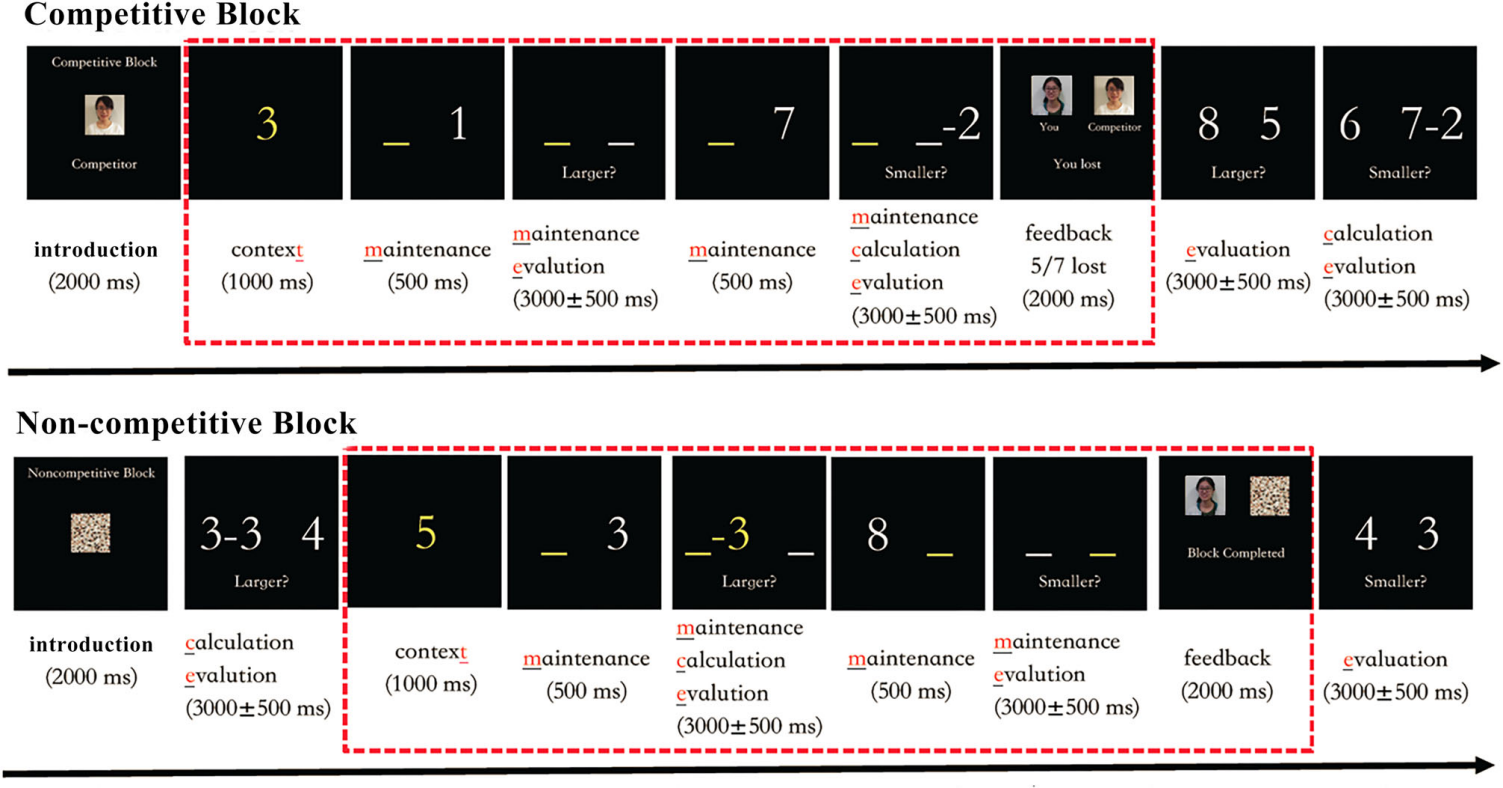
The event-related numerical WM task paradigm.

We applied a new event-related “number calculation working memory” task from previous work, which comprised alternating competitive and non-competitive blocks (NumComp-task) [23] (Fig. 1). The participants received task training before MRI scanning to ensure that they understood the task well.

During the trials with competition (C), participants were led to believe that they were playing a number calculation game against a competitor of similar age and gender, and were judged based on timing and accuracy. The NumComp-task had two sessions: 14 competitive trials and 14 non-competitive trials in total. To induce social stress, participants received more negative feedback of “you lost” (5/7) among the 14 competitive trials. During the non-competitive (NC) trials, the participants played the game with no competitor and received a neutral response.

For each trial under the WM conditions, the participants were presented with two numbers from 1 to 9 successively to encode into WM and subsequently had to be maintained. Then they were asked which of the two results was “larger” or “smaller” as instructed (maintenance evaluation, ME) or had to perform mental arithmetic (subtraction) on one of the numbers before giving a response to the “larger” or “smaller” comparison (maintenance calculation evaluation, MCE). Further details of the NumComp-task are available in the supplementary material.

The NumComp-task had competitive and non-competitive blocks. For WM (enclosed in red dashes), participants encoded 2 integer numbers in yellow or white presented over 1 s and to be retained in WM (underlined in the same color, yellow or white) across an interval of 3–5 s. In maintenance trials, participants responded to which of the two numbers was “larger” or “smaller” within 2.5–3.5 s. In the manipulation trials, participants had to do mental arithmetic on one of the two numbers in yellow or white before the “larger” or “smaller” evaluation within 2.5–3.5 s. These two kinds of trials were embedded within equal numbers of competitive or non-competitive blocks. Two blocks were counterbalanced in 2 runs. Each run took ~ 10 min. All the instructions were originally in Chinese. All “competitors” were of the same gender and age. The interspersed fixation was between each complete competitive block and a noncompetitive block and was not shown.

### Image Acquisition

Imaging data were acquired using a 3.0-Tesla GE scanner (Discovery MR750) at the Center for Neuroimaging, Peking University Sixth Hospital. Foam pads were used to minimize head motion. A gradient echo sequence was used to acquire blood oxygen level-dependent (BOLD) functional MRI (fMRI) images, and each volume consisted of 33 axial slices covering the entire cerebrum and cerebellum with the following parameters: thickness/gap = 4.2 mm/0 mm, TR/TE = 2000/30 ms, flip angle = 90°, field of view = 22.4 cm × 22.4 cm, matrix = 64 × 64. Three dummy scans were acquired at the beginning of the fMRI scanning.

### Image Processing and Analyses

We excluded participants with an accuracy rate < 50% in any WM conditions (8 OCDs), those with head motion > 3 mm translation or 3° rotation (8 OCDs and 6 HCs), and those with image artifacts or did not complete the task (4 OCDs and 6 HCs) for quality control. In total, 93 participants (38 OCDs and 55 HCs) were included in the final analysis (Table 1).

The fMRI imaging data were pre-processed using MatLab 2016b and SPM12 (http://www.fil.ion.ucl.ac.uk/spm). Functional images for each participant was slice-timing corrected, realigned to the first volume in the time series, and corrected for head motion. Images were then spatially normalized into standard stereotaxic space (Montreal Neurological Institute template) using fourth-degree B-spline interpolation. Spatial smoothing was applied with a Gaussian filter set at 8 mm full-width at half-maximum. Each task-evoked stimulus was modelled as a separate delta function and convolved with a canonical hemodynamic response function; ratio normalized to the whole-brain global mean to control for systematic differences in global activity, and temporally filtered using a high-pass filter of 128 s. Each task-evoked stimulus event was modelled for correctly-performed trials in the general linear model of the first-level analysis of the image data. Incorrect responses and residual movement parameters were also modelled as regressors of no interest [26].

The planned contrasts of interest for a second-level random effect within-group analysis using one-sample t-tests were brain activity in the maintenance (ME) or manipulation (MCE) task conditions under non-competitive (NC) and competitive (C) condition at *P *< 0.05, whole-brain family-wise error (FWE) corrected.

For the diagnosis effect (group difference) on brain activity in each WM condition (ME or MCE) under competitive or non-competitive condition, the significance level was set as an uncorrected *P* < 0.001, with a cluster sizes ≥ 86 voxels (688 mm^3^) for ME_C, ≥ 94 voxels (752 mm^3^) for ME_NC, ≥ 83 voxels (664 mm^3^) for MCE_C, and ≥ 103 voxels (824 mm^3^) for MCE_NC with independent-sample *t*-tests, which corresponded to a corrected *P* < 0.05 determined by 5000 Monte Carlo simulations using AlphaSim correction within the grey matter mask in DPABI_V4.0 (http://rfmri.org/dpabi). The inter-subject variability was treated as a random effect, controlled for age, gender, and years of education.

For the stress effect indicated as brain activity during the maintenance or manipulation task conditions under competition *versus* non-competition in the HC and OCD groups, the significance level was set as an uncorrected *P* < 0.001, with cluster sizes ≥ 125/124 voxels (1000/992 mm^3^) for HC and ≥ 137/229 voxels (1096/1832 mm^3^) for OCD with one-sample *t*-tests, which corresponded to a corrected *P *< 0.05 determined by 5000 Monte Carlo simulations using AlphaSim correction within the grey matter mask in DPABI_V4.0 (http://rfmri.org/dpabi). The imipramine equivalent was included as a covariate to control for the medication effect in the OCD within-group analysis.

The contrast images of the competitive or non-competitive condition were subsequently subjected to a flexible 2 × 2 analysis of variance (ANOVA) to explore the brain activity of the diagnosis × stress interaction effect separately in the ME and MCE conditions. The significance level was set at an uncorrected *P* < 0.001, with cluster sizes ≥ 38 voxels (304 mm^3^) for maintenance and ≥ 95 voxels (760 mm^3^) for manipulation, which corresponded to a corrected *P* < 0.05 determined by 5000 Monte Carlo simulations using AlphaSim correction within the grey matter mask in DPABI_V4.0 (http://rfmri.org/dpabi). Around the peak coordinates, an 8 mm radius sphere was created using DPABI_V4.0 as the region of interest (ROI) of each area showing a significant interaction effect, and then the contrast values of the ROIs from the corresponding contrast images of each task and condition in each group were extracted for a two-way repeated measures ANOVA and a simple main effect analysis using GraphPad Prism 7.0 (https://www.graphpad.com). The significance level of the ANOVA and simple main effect analysis in GraphPad Prism 7.0 was set at *P* < 0.05, false discovery rate (FDR) correction.

### Statistical Analyses of Clinical and Behavioral Data

Demographic and clinical data were analyzed with a standard statistical package (IBM SPSS 21.0, Chicago, IL), using the *t*-test and *χ*^2^ test. Task behavioral data (accuracy rate and reaction time) of the two groups under stress and non-stress condition were analyzed with GraphPad Prism 7.0 using two-way repeated measures ANOVA. If the diagnosis × stress interaction effect was significant, a simple main effect analysis was applied as a *post hoc* analysis in GraphPad Prism 7.0. If the brain activity of diagnosis × stress interaction effect was significant in ME or MCE conditions, the stress-related contrast values of the ROI of the interaction effect from the corresponding contrast images of each task condition (ME or MCE under competitive *versus* non-competitive contrast images) in the OCD group were extracted and correlated to the clinical variables (Y-BOCS, HAMA, and HAMD) and task behavioral performance. The significance level of the *t*-test and *χ*^2^ test was set at *P* < 0.05. The significance level of the two-way repeated measures ANOVA, simple main effect, and correlation analysis was set at *P* < 0.05, with FDR correction.

## Results

### Behavioral Performance

**Fig. 2:**
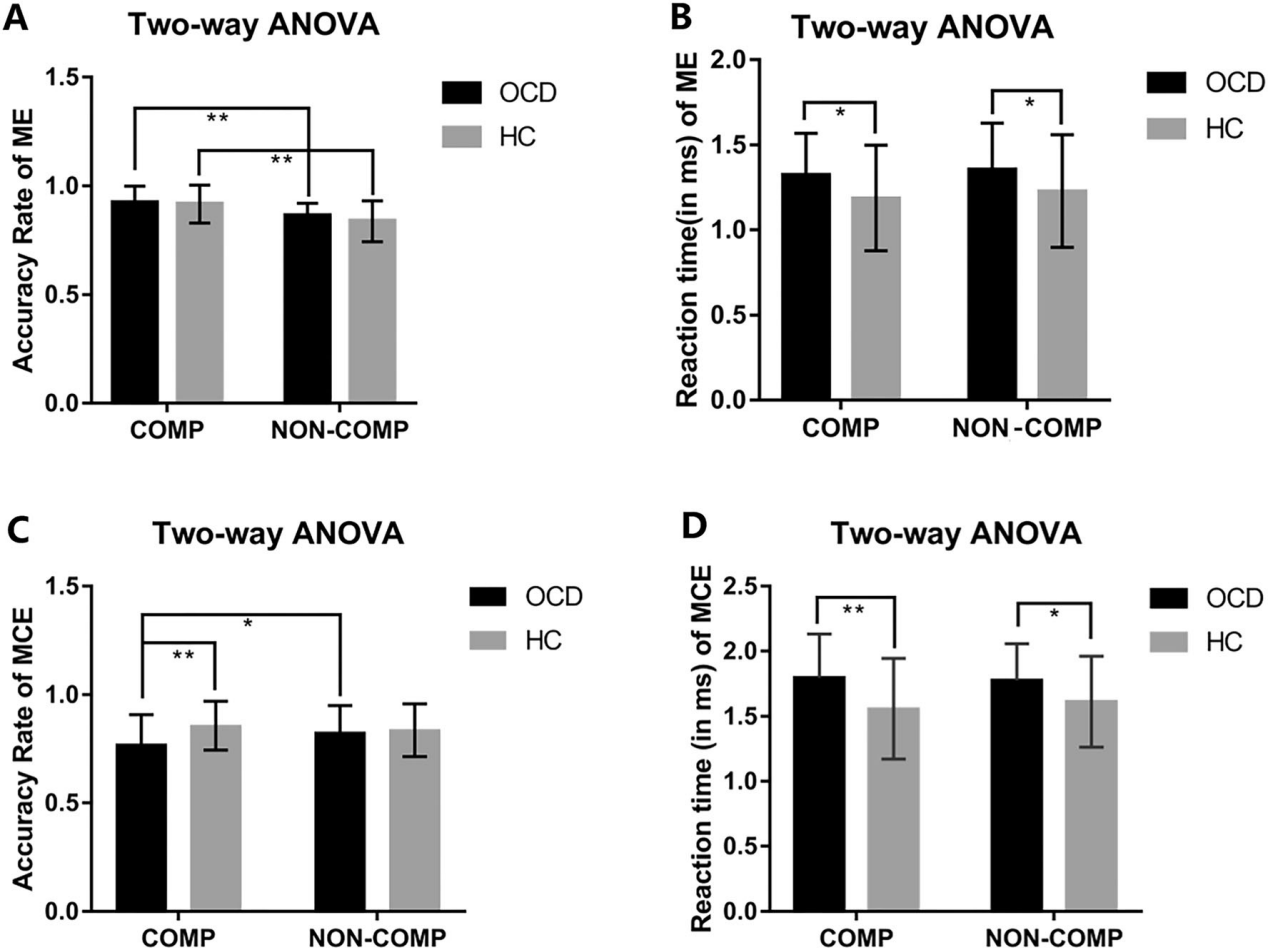
Results of two-way ANOVA of diagnosis and stress and multiple comparisons in behavioral performance. **A** Stress has the main effect on accuracy rate in the ME task. The accuracy rate is higher under competition than non-competition in both groups. **B**, **D** Diagnosis has the main effect on average reaction time in the ME and MCE tasks. The average reaction time is longer in OCDs than HCs under both competitive and non-competitive conditions. **C** Stress and diagnosis have an interaction effect on accuracy rate in the MCE task. The accuracy rate is lower in OCDs than HCs only under the competitive condition and the accuracy rate is lower under competitive than non-competitive conditions only in OCDs. ME, maintenance evaluation; MCE, maintenance calculation evaluation; C, competition; NC, non-competition *P < 0.05, **P < 0.01.

In terms of the accuracy rate of maintenance, we only found significant main effects of stress (competitive *vs* non-competitive, *F =* 38.47, *P *< 0.0001). The accuracy was higher during the competitive than the non-competitive condition in OCDs (*t =* 3.48, *q =* 0.0008, FDR correction) and HCs (*t =* 5.52, *q* < 0.0001, FDR correction) (Fig. 2A and Table S2). As for the accuracy rate in the manipulation task, the main effect of diagnosis (OCD *vs* HC, *F =* 4.89, *P =* 0.03) and the interaction effects of diagnosis × stress (*F =* 7.47, *P =* 0.008) were significant. The accuracy was lower in OCDs than in HCs only during the competitive condition (*t =* 3.32, *q =* 0.001, FDR correction) and the accuracy was lower under the competitive than the non-competitive condition only in OCDs (*t =* 2.57, *q =* 0.01, FDR correction) (Fig. 2C and Table S2–S3).

In terms of reaction time in the maintenance and manipulation tasks, the main effects of diagnosis (*F =* 4.87, *P =* 0.03, ME; *F =* 8.54, *P =* 0.004, MCE) and stress (*F =* 5.02, *P =* 0.03, only in ME) were significant and there was no significant diagnosis × stress interaction effect (Table S2). The reaction time was longer in OCDs than that in HCs during both the competitive (*t =* 2.20, *q =* 0.043, FDR correction, ME; *t =* 3.31, *q =* 0.002, FDR correction, MCE) and non-competitive (*t =* 2.06, *q =* 0.043, FDR correction, ME; *t =* 2.27, *q =* 0.03, FDR correction, MCE) conditions. The reaction time was shorter under the competitive than the non-competitive ME condition. However, there was no significant stress effect within each group (Fig. 2B and Fig. 2D).

### Brain Activation

#### WM-Related Brain Activation

**Fig. 3:**
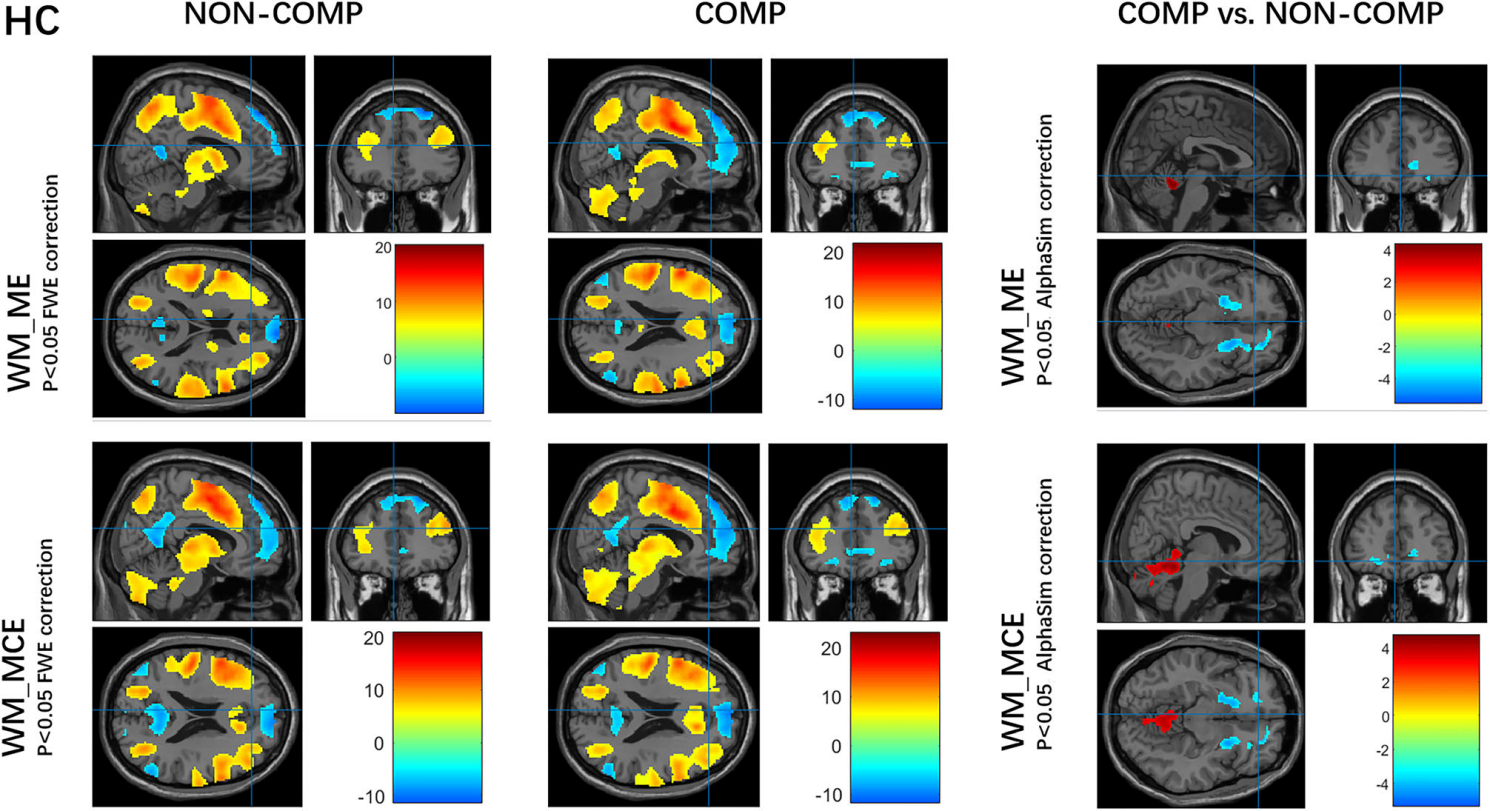
Working memory-related brain activity and stress effect in the HC group. **A** Activity in the working memory task under competition and non-competition (left, *P *< 0.05, FWE correction, cluster size > 5 voxels). **B** Activity of competitive *versus* non-competitive conditions group under different task patterns (right, *P *< 0.05, AlphaSim correction for multiple comparisons). ME, maintenance evaluation; MCE, maintenance calculation evaluation; COMP, competition; NON-COMP, non-competition; FWE, whole-brain family-wise error corrected.

**Fig. 4:**
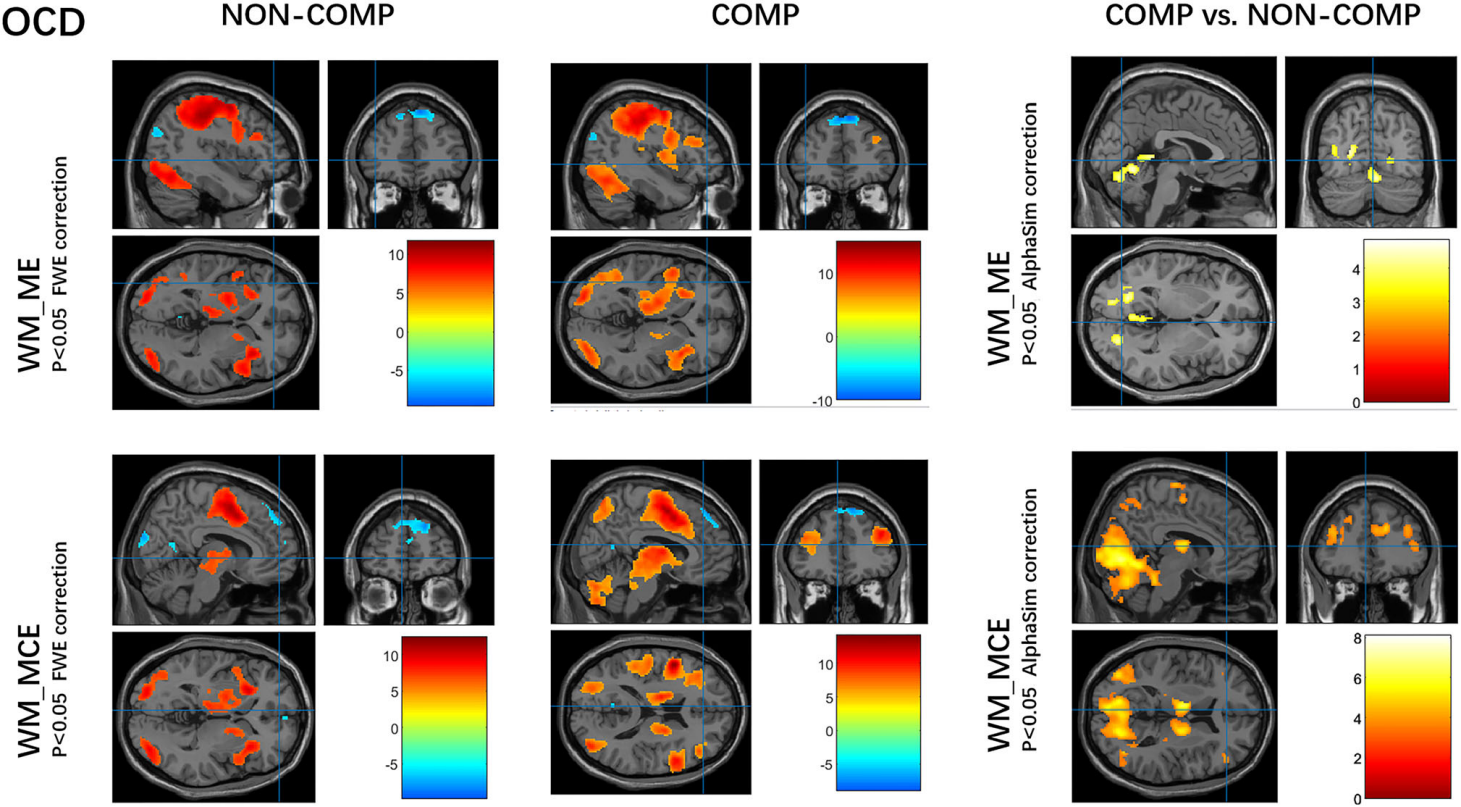
Working memory-related brain activity and stress effect in the OCD group. **A** Activity of working memory task under competition and non-competition (left, *P *< 0.05, FWE correction, cluster size > 5 voxels). **B** Activity of competitive *versus* non-competitive conditions under different task patterns (right, *P *< 0.05, AlphaSim correction for multiple comparisons). ME, maintenance evaluation; MCE, maintenance calculation evaluation; COMP, competition; NON-COMP, non-competition; FWE, whole-brain family-wise error corrected.

During each of the WM maintenance and manipulation tasks under the competitive or non-competitive condition in both OCDs and HCs, regions in the prefrontal, parietal, temporal, occipital, and cerebellar cortices, and the striatum were robustly activated, along with well-established deactivation in areas of the default mode network (DMN) during the cognitive task, including the mPFC and posterior cingulate cortex (*P *< 0.05, whole-brain FWE correction for multiple comparisons, see Figs. 3 and 4, left).

There was no significant between-group difference in the brain activity in WM maintenance or manipulation under competitive and non-competitive conditions.

### Stress Effect on Brain Activity in Each Group

In terms of stress effects on WM maintenance and manipulation, a pattern of less engagement of the basal ganglia (less activation) and mPFC (more deactivation), more activation of the cerebellum was found during maintenance and manipulation under competitive *versus* non-competitive conditions in HCs (*P *< 0.05, AlphaSim correction for multiple comparisons, see Fig. 3, right, and Table S4).

The pattern of reduced engagement of the basal ganglia and mPFC in HCs was absent from the OCDs. Taking the WM manipulation for example, the more activated regions included the dorsal and ventral anterior cingulate, superior temporal lobe, bilateral hippocampus, bilateral thalamus, and bilateral SMA which are implicated in the neural processing of goal-directed behavioral control, habit learning, and stress; while the less deactivated regions included the medial frontal gyrus and bilateral temporal cortex, which are implicated in the DMN (*P *< 0.05, AlphaSim correction for multiple comparisons, see Fig. 4, right, and Table S5).

### Diagnosis and Stress Interaction Effects

**Fig. 5:**
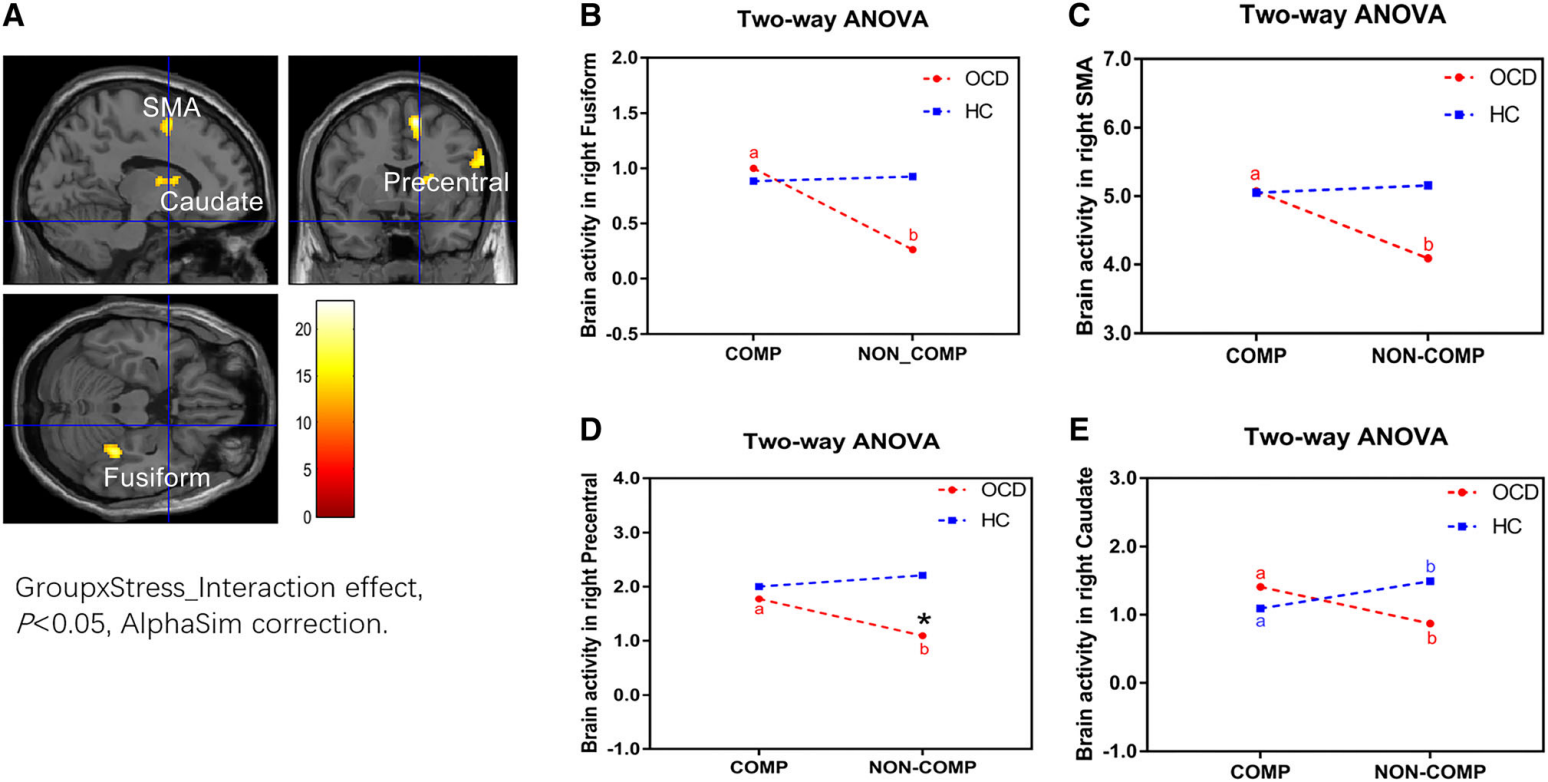
Interaction effect of diagnosis and stress in brain areas. **A** The right fusiform, supplementary motor area, precentral, and right caudate are areas of the diagnosis and stress interaction effect. **B**–**E** Activation of the right fusiform (**B**), supplementary motor area (**C**), precentral (**D**), and right caudate (**E**) is higher under competitive than non-competitive condition only in OCDs; a, b, activation of the right caudate is lower under competitive than non-competitive conditions only in HCs; *under the non-competitive condition, the BOLD signal in the right precentral is higher in HCs than OCDs.

For the WM manipulation task, we found a significant diagnosis × stress interaction effect in the right fusiform cortex, right supplementary motor area (SMA), right precentral cortex, and right caudate (*P *< 0.05, AlphaSim correction for multiple comparisons, Fig. 5A and Table 2). The contrast values of the four ROIs in WM manipulation under competitive or non-competitive conditions of each group were extracted for further analysis. The activation of all four areas was significantly higher under competitive than non-competitive conditions only in OCDs (fusiform, *t =* 4.66, *q* < 0.0001; SMA, *t =* 4.63, *q* < 0.0001; precentral cortex, *t =* 4.37, *q* < 0.0001; caudate, *t =* 2.83, *q =* 0.01; FDR correction; Fig. 5B–E and Tables S6–S10). The activation of the right caudate was lower under competitive *versus* non-competitive conditions only in HCs (*t =* 2.53, *q =* 0.01, FDR correction; Fig. 5E and Table S10). In conclusion, the stress effect on brain activity differed between OCDs and HCs. The right fusiform, SMA, precentral cortex, and caudate had increased stress-related activity in OCDs, but not in HCs, while the right caudate had decreased stress-related activity in HCs.

**Table 2:** The diagnosis × stress interaction effect in brain areas in the manipulation task (MCE) (*P *< 0.05, AlphaSim correction).

Variables	Cluster Size	Structure (aal)	Peak MNI coordinates			Peak intensity
	130	Fusiform_R (aal)	32	− 36	− 20	4.36
	158	Supp_Motor_Area_R (aal)	8	4	56	4.34
	164	Precentral_R (aal)	62	4	28	3.96
		Precentral_R (aal)	54	4	24	3.67
	227	Caudate_R (aal)	18	18	2	3.79
		Caudate_R (aal)	14	10	12	3.69
		Caudate_R (aal)	14	0	12	3.62

No significant brain activity of diagnosis × stress interaction effect was found for WM maintenance.

### Correlation Analysis

Correlation analyses were carried out between the stress-related activity of the brain regions with a significant diagnosis × stress interaction and the stress-related behavioral measures in OCDs. Considering the stress effect was only significant in accuracy rate but not in reaction time in OCDs (Fig. 2), the measure of the task performance only included the accuracy rate difference between manipulation under competitive and non-competitive condition.

In the OCD patients, the severity of obsessions and compulsions was positively correlated with their anxiety and depression symptoms (Table S11). Behavioral performance of the maintenance and manipulation tasks was significantly correlated with symptom severity (worse symptoms of depression correlated with faster responses), and more severe obsession symptoms were associated with a lower accuracy rate: obsession severity factors were negatively correlated with the accuracy rate in the manipulation task under stress (*r =* –0.479, *P* < 0.01; *r =* –0.358, *P* < 0.05) (Table S12).

In terms of the correlation between the neural activation of the stress effect with the symptoms and task performance, only significantly negative correlations were found with the clinical symptom severity (Table S13), such as a stress-related activation change in the right fusiform and SMA showing negative correlations with the obsession interference score and time score, respectively (*r =* –0.465, *P* < 0.01; *r =* –0.353, *P *< 0.05); and no significant correlation with the accuracy rate difference.

However, no correlation survived the FDR correction.

## Discussion

We investigated the effect of stress on the neural processing associated with cognitive changes of WM in OCD and its relationship with the clinical variables. Stress impaired the WM behavioral performance in OCDs but not in HCs, specifically in more difficult WM manipulation. OCD patients showed a WM activation pattern similar to that of HCs: increased WM-related activity in the prefrontal-parietal-striatum network and decreased activity in the DMN, consistent with previous studies in HCs [22, 26–28]. The stress effect on the WM-related activity pattern in OCDs (a failure to suppress the DMN) differed from HCs (more deactivation in the mPFC along with less activation in the striatum). The diagnosis effect under each task condition (ME or MCE) and each stress condition (competitive or noncompetitive) on the activity was not significant, while the diagnosis and stress interaction effect on activation was significant in the right fusiform, SMA, precentral cortex, and right caudate only in the WM manipulation. OCD patients had stress-related hyperactivity in the right fusiform, SMA, precentral cortex, and right caudate, and HCs had stress-related suppression in the right caudate. Further, the clinical symptoms in OCD were associated with their task performance and the stress-related changes of brain activation.

### Task Performance and Stress Effect

OCD patients performed worse than HCs but this was only indicated by the longer reaction time in WM maintenance and manipulation and worse accuracy in WM manipulation under stress. Under stress, the task performance was improved in WM maintenance and intact in the more difficult WM manipulation in HCs, while in OCDs under stress, the performance was also improved in WM maintenance but impaired in WM manipulation. Previous meta-analysis of the neuropsychology of OCD only found small negative mean effect sizes for WM across studies, which suggested that OCD patients perform worse than controls to a relatively small extent [9]. Although few previous studies have explored the role of acute stress in WM in OCD patients, several studies of HCs have shown that stress tends to increase the accuracy of WM [29, 30]. A previous meta-analysis implied that the overall influence of acute stress on WM is deleterious, depending on stress severity, WM load, and percentage of male participants [31]. Although no significant between-group difference in the brain activity was found in WM maintenance or manipulation under stress and non-stress, the behavioral findings in the current study suggested that WM in patients with OCD was impaired to a less severe extent, consistent with the previous findings, and might be more vulnerable to stress than HCs.

### Brain Activation

#### Less Engagement of the mPFC and Limbic System in HCs Under Stress

In the HCs, the stress effect on WM activations included less involvement of the mPFC (more deactivation) and the striatum (less activation) and increased activity in the cerebellum. The finding of less activation in the striatum is consistent with previous work on HCs in which stress increased activity in prefrontal and parietal cortex [29] and reduced activity in limbic areas including the ventral striatum, hypothalamus, amygdala, and hippocampus [32]. Moreover, Kogler *et al*. found psychosocial stress-related deactivation in the striatum and activation in the superior temporal cortex in a meta-analysis of neuroimaging studies [33]. The mPFC plays an important role in processing information about stressors and regulating the corresponding behavioral responses [34–36]. Van Leeuwen *et al*. investigated the responses to an emotional stimulus after inducing acute stress in healthy adults and the healthy siblings of patients with schizophrenia. They found that the HCs, but not the siblings of schizophrenics, showed reduced deactivation in the mPFC, middle cingulate gyrus, posterior cingulate gyrus, precuneus, and superior temporal cortex following stress; these were consistent with the regions of the DMN [37]. Thus, we assumed that suppression of the mPFC might be a reassignment of neural resources to the stress response by suppressing self-referential processes and salience detection as well [37]. However, conflicting studies have found that, after inducing stress, the dorsolateral PFC is less activated and the medial orbitofrontal cortex within the DMN is less deactivated than under the non-stress condition [38, 39]. The reason could be that an overly strong stress induced higher cortisol levels or other possible confounding factors that made the finding different from the previous [29] and our findings.

### Failure to Suppress the Default Mode Network in OCD Patients Under Stress

OCD patients showed a pattern different from the HCs: regions in the DMN, including the mPFC and bilateral temporal cortex were more involved (less deactivation) under competitive *versus* non-competitive conditions. A recent study investigated the deactivated pattern of the DMN in OCDs and HCs under a transition from a resting to a non-resting context. They indicated that OCDs had a failure to suppress the DMN activity even during the emotion-provoking stimulus [40]. Using the same MIST task, Lord *et al*. reported less deactivation in the mPFC and orbitofrontal cortex induced by stress in postpartum OCD patients [41]. A deficit of DMN suppression has also been found in schizophrenia and depression during WM tasks [42–44], and failure of the DMN suppression is associated with a worse behavioral performance in schizophrenia and depression [42]. Thus, less suppression of the DMN under stress could be a common neural response to stress in OCD, schizophrenia, and depression and might be correlated with worse behavioral performance.

Previous studies have generally suggested that the DMN supports an internal model for the self-referential process [45, 46]. DMN suppression plays an important role in external goal-directed cognition tasks by suppressing certain internal activity such as daydreaming and self-referential thought [42]. A study of large-scale brain networks considered that the networks are dynamically-shifting and resource re-allocated by neurobiological modulators to adapt to acute stress [20]. Previous functional connectivity studies of OCD reported dysconnectivity within the DMN and between networks for salience, frontoparietal and the DMN [47], and reduced connectivity within DMN subsystems [48]. Therefore, a failure of DMN suppression during cognition and under stress might be one of the neural mechanisms underlying the cognitive impairment induced by stress in OCDs.

### Stress-Related Hypersensitivity in OCD

An interaction effect of diagnosis and stress was found both on behavioral performance and neural response only in WM manipulation, indicating that WM deficits in OCD are load-dependent and can be influenced by stress. Activations in the right fusiform, SMA, precentral cortex, and right caudate were significantly higher in the OCD group after inducing stress.

#### The Fusiform Gyrus

The lateral part of the fusiform gyrus was named as the fusiform face area (FFA) because of its specific role in face processing [49]. Few studies have investigated the function of the FFA in OCD patients. However, increased fMRI activity in the FFA has been found in schizophrenia to compensate for the damaged basic integration capability during spatial frequency-degraded face processing [50]. The FFA is hyperactivated and has greater connectivity with the amygdala in social anxiety disorder when handling the fearful face [51]. Moreover, several previous studies in healthy adults have also found interpersonal competition-related hyperactivation in the right fusiform and suggested its role in the social interaction process [52–54]. Thus, we assumed that the hyperactivity of the right fusiform in OCD was compensation to process the relevant information of the WM task under interpersonally competitive stress.

#### The Supplementary Motor Area

The right SMA showed greater activation after inducing stress in OCD patients under the manipulation task and was negatively correlated with the severity of obsession. De Vries *et al*. reported hyperactivity of the pre-SMA in OCD patients during a higher-level n-back task [17] and a response inhibition task [55]. They also found that right pre-SMA activation is negatively correlated with illness severity, suggesting that hyperactivity of the pre-SMA is a candidate endophenotype of OCD. The SMA, pre-SMA and the supplementary eye field compose the supplementary motor complex (SMC), which links cognition to action [56]. Studies of macaque monkeys has provided the most evidence for the anatomy and connections between the SMC and the basal ganglia (including direct, indirect, and hyper-direct pathways similar to the CSTC circuit) [56]. Lesion studies of the SMC reported difficulty in task-switching that resulted in compulsivity [57, 58]. Thus, we assumed that the SMC takes part in the neuropathological mechanism of OCD and is activated in compensation for the stress response.

#### The Precentral Gyrus

The primary motor cortex is included in the precentral gyrus and is responsible for motor responses. Previous studies have found hyperactivity of the precentral gyrus in emotional regulation-related tasks in mood and anxiety disorders and high-risk individuals [59–61]. Thus, the finding of significantly lower activation of the precentral gyrus in OCDs compared with HCs under non-competitive conditions but significant hyperactivity after inducing stress in OCDs is consistent with the results of previous research and might be a compensatory mechanism for stress response.

#### The Caudate

Caudate dysfunction is one of the most consistent findings in OCD. Previous work on the brain morphology of OCD patients consistently found increased volume in the caudate [62, 63]. Resting-state functional connectivity (FC) studies of OCD patients found that the decreased FC between the ventrolateral PFC and the caudate is associated with decreased cognitive flexibility [64]. A symptom provocation study reported reduced activation in the caudate nucleus [65], while activation of the caudate is considered to contribute to goal-directed behavior [66, 67]. Few previous studies have explored stress-related activation of the subcortical regions during WM in OCD patients. However, several studies have reported stress-related hyperactivity in the caudate in depressed patients, reflecting hypersensitivity to stress [44, 68]. Thus, according to the pathological model of OCD, acute stress might induce the compensatory upregulation of activity in the caudate to increase goal-directed action and to improve WM performance in OCD.

The four brain regions discussed above are associated with the pathological circuit of OCD [56, 69] and might also be involved in social stress processes [70]. Thus, we assumed that their activation is compensatory upregulation for the stress response. However, more research is needed to verify the existence of an OCD-specific stress network.

### Clinical Implications

The accuracy rate was negatively correlated with the OCD symptom severity score, especially the accuracy rate in WM manipulation under stress, and the reaction time of all task conditions was negatively correlated with the scores on the HAMA and HAMD, which indicated that neural dysfunction related to the psychopathology of OCD is associated with the WM network dysfunction. The previous meta-analysis of resting-state fMRI showed that the frontoparietal regions are the commonly impaired circuits in both the assumed OCD model and executive and WM networks [36, 47], consistent with the behavioral findings in the current study. The contrasts of compensatory upregulation of the four areas were negatively correlated with OCD symptom severity, which indicate that patients with less severe symptoms are better able to compensate.

## Limitations and Future Directions

This study has several potential limitations. First, given the limited sample size, we could not further explore the stress effect on different subtypes of OCD or gender to investigate the relationship between clinical heterogeneity and neural responses to stress. Second, more than half of the patients were taking antidepressants during fMRI scanning. Although we included medication dosage as confounding, it may still have influenced brain activity. It is necessary to recruit drug-naive patients in future. Third, the OCD and HC groups were well matched in demography but for age. However, we tried to remove the linear effect from all the findings and most of the participants in both groups were in their twenties. Therefore, age was less likely to be the main factor causing a between-group difference. Nevertheless, a future study with a larger sample of OCD patients and including other neuropsychiatric disorders is necessary to verify the current findings and explore their specificity over diseases. Fourth, the relationship between altered neural activity and symptoms did not conform to the expected direction, although some previous study have reported similar findings [55]. Besides, no correlation findings survived FDR correction in the current study. Therefore, we should treat all the correlation findings with caution before they are verified with further evidence.

## Conclusion

The study provides evidence that OCD patients are more vulnerable to acute stress, which affects their WM-related neuro-mechanisms. The failure of suppression of the mPFC and striatum and stress-related hyperactivation of the right fusiform, SMA, precentral, and right caudate might be OCD-related psychopathological and neural responses to stress.

## Electronic supplementary material

Below is the link to the electronic supplementary material.

Supplementary material 1 (PDF 118 kb)
