# Supplementary material for: Distinct Effects of Social Stress on Working Memory in Obsessive-Compulsive Disorder
Source: Neurosci Bull. 2020 Oct 1;37(1):81–93. doi: 10.1007/s12264-020-00579-3 (PMC7811969; doi:10.1007/s12264-020-00579-3)
Supplement: Supplementary file 1 — Supplementary material 1 (PDF 118 kb) [file 12264_2020_579_MOESM1_ESM.pdf]

Supplementary Materials

The "number calculation working memory" task

The task paradigm was written with Presentation software (Neurobehavioral Systems, Inc., Berkeley, CA). Visual stimuli were projected onto a screen placed behind the subject's head and reflected to the subject by a mirror over the head coil. The subject held a response handle with two buttons in the right hand. The MRI scanning was triggered by the task presentation. All subjects were trained before scanning to make sure that they understood the task well, and their response time and accuracy rate were recorded during the scanning.

The "number calculation working memory" task (NumComp-task) had two sessions, each lasting ~10 min, in which 14 trails were under competitive and 14 trails under non-competitive conditions. Figure 1 shows a competitive block and a noncompetitive block, including introduction, task response phase, and feedback. Positive feedback was given as "You Win" and negative as "You Lost" in the competitive trails, during which subjects were led to believe that they were playing the game against a competitor of similar age and gender, and were judged based on timing and accuracy rate. To induce social stress, subjects received more negative feedback of "you lost" (5/7) among the 14 competitive trails. Neutral feedback was given in the non-competitive trails as "Block Completed". The interspersed fixation was between each complete competitive block and a noncompetitive block.

The task response phase included 2 WM trials. As presented in Figure 1, the dotted boxes in red indicate a complete block of WM trials. The subjects encoded 2 integer numbers in yellow and white presented over 1 s to be retained in WM (underlined in the same color) across an interval of 3–5 s. In the maintenance (ME) trials, subjects responded to which of the two numbers was "larger" or "smaller" within 2.5–3.5 s, while in the manipulation (MCE) trials, subjects had to do mental arithmetic on one of the two numbers in yellow or white before the "larger" or "smaller" evaluation within 2.5–3.5 s.

Supplementary Tables

Table S1. Factor scores of the Yale-Brown scale in OCD patients

Variables	OCD<br>(*n* = 38)*
YBOSC Factor	
Symmetry	2.8±2.0
Taboo thoughts	4.7±3.8
Contamination	5.6±4.2
Hoarding	0.8±0.9
YBOSS Factors	
Time (spent on or occupied with symptoms) _Obsession	2.0±0.8
Interference (with functioning or relationship) _Obsession	2.1±0.9
Distress (associated with the symptoms) _Obsession	2.4±0.8
Resistance (to the symptom) _Obsession	2.1±1.3
Control (of the symptom) _Obsession	2.5±1.0
YBOSS _Obsession	11.2±3.7
Time (spent on or occupied with symptoms) _Compulsion	2.1±1.0
Interference (with functioning or relationship) _Compulsion	2.1±1.2

Distress (associated with the symptoms) _Compulsion	2.5±1.3
Resistance (to the symptom) _Compulsion	2.5±1.3
Control (of the symptom) _Compulsion	2.6±1.1
YBOSS _Compulsion	11.8±4.7

YBOSC, Yale-Brown Symptom Checklist; YBOSS, Yale-Brown severity scale; YBOSS _Obsession: total obsession score in Yale-Brown severity scale; YBOSS _Compulsion: total compulsion score in Yale-Brown severity scale. *unless otherwise indicated, data are the mean ± standard deviation.

Table S2. Two-way ANOVA of accuracy rate and average reaction time under different task patterns

Source	Type III Sum of Squares	df	Mean Square	*F*	*P* value
**Variable: Accuracy rate of ME**					
Diagnosis	0.01	1	0.01	1.36	0.25
Stress	0.22	1	0.22	38.47	**<0.0001**
Diagnosis × Stress	0.004	1	0.004	0.72	0.40
**Variable: Accuracy rate of MCE**					
Diagnosis	0.11	1	0.11	4.89	**0.03**
Stress	0.01	1	0.01	1.50	0.22
Diagnosis × Stress	0.06	1	0.06	7.47	**0.008**
**Variable: Average reaction time of ME**					
Diagnosis	0.80	1	0.80	4.87	**0.03**
Stress	0.06	1	0.06	5.02	**0.03**
Diagnosis × Stress	0.0009	1	0.0009	0.07	0.79

**Variable: Average reaction time of MCE**					
Diagnosis	1.85	1	1.85	8.54	**0.004**
Stress	0.01	1	0.01	0.57	0.45
Diagnosis × Stress	0.07	1	0.07	3.08	0.08

ME, maintenance evaluation; MCE, maintenance calculation evaluation.

Table S3. Differences in accuracy rate under the manipulation task

Variables	OCD	HC	*t*	*q* Value (FDR)
COMP_ Accuracy	0.72±0.18	0.86±0.11	3.32	**0.001**
NON-COMP _Accuracy	0.78±0.18	0.84±0.12	0.43	0.35
Variables	COMP	NON _COMP	*t*	*q* Value (FDR)
OCD_ Accuracy	0.72±0.18	0.78±0.18	2.57	**0.01**
HC_ Accuracy	0.86±0.11	0.84±0.12	1.18	0.12

COMP, competition; NON-COMP, noncompetition; q Value (FDR), multiple comparisons after two-way ANOVA, FDR-corrected.

Table S4. Stress-related activity changes in the HC group (*P* <0.05, AlphaSim correction)

Variables	Cluster size	Structure (aal)	Peak MNI coordinates			Peak Intensity
**HC_ME C>NC**						
	139	Vermis_3 (aal)	-2	-46	-20	4.30
**HC_ME C<NC**						
	1151	Putamen_R (aal)	24	14	-2	5.50
		Frontal_Inf_Orb_R (aal)	24	28	-14	4.84
		Middle Frontal Gyrus	20	46	-10	4.63
	477	Extra-Nuclear_L	-26	6	-10	4.67
		Olfactory_L (aal)	-20	8	-16	4.42
		Extra-Nuclear _L	-20	20	-8	4.14

**HC_MCE C>NC**					
1509	Cerebellum_4_5_L (aal)	-2	-56	-14	4.73
	Vermis_3 (aal)	0	-48	-18	4.53
	Vermis_6 (aal)	0	-72	-18	4.28
**HC_MCE C<NC**					
586	Putamen_R (aal)	22	14	-2	5.31
	Rectus_R (aal)	20	12	-14	4.44
397	Brodmann area (BA) 13	-18	14	-12	4.58
	Putamen_L (aal)	-24	4	-8	4.53
	Putamen_L (aal)	-22	18	0	4.39
167	Superior Frontal Gyrus_R	18	48	-10	4.01
	Superior Frontal Gyrus_R	22	52	-2	3.92

	Frontal_Sup_Medial_R (aal)	16	46	0	3.87

ME, maintenance evaluation; MCE, maintenance calculation evaluation; C, competition; NC, noncompetition.

Table S5. Stress-related activity changes in the OCD group (*P* <0.05, AlphaSim correction)

Variables	Cluster size	Structure (aal)	Peak MNI coordinates			Peak intensity
**OCD_ME C>NC**						
	731	Lingual_L (aal)	-26	-64	-4	4.85
			-30	-64	4	4.84
		Temporal Lobe_L	-24	-62	16	4.6
	629	Vermis_4_5 (aal)	-2	-60	-8	4.76
		Vermis_6 (aal)	4	-74	-18	4.57
		Corpus Callosum	-2	-40	4	4.28
	146	Lingual_R (aal)	20	-76	0	4.28
**OCD_MCE C>NC**						
	20694	BA 18/Lingual_R (aal)	16	-76	-2	8.09
		Lingual_L (aal)	-8	-74	-4	6.69
		Lingual_L (aal)	-12	-82	0	6.66
	747	BA 6/Frontal_Sup_R (aal)	18	16	60	6.4
		BA 6/Supp_Motor_Area_R	12	8	58	6.01

	(aal)				
	Frontal_Sup_R (aal)	20	12	50	4.53
392	Superior Temporal Gyrus_R	42	6	-18	5.26
	Superior Temporal Gyrus_R	42	18	-18	4.57
	Temporal_Pole_Sup_R (aal)	32	12	-24	4.34
384	BA 6/Frontal_Mid_R (aal)	44	-2	56	4.95
	Precentral_R (aal)	28	-6	52	3.98
	Precentral_R (aal)	32	-16	58	3.79
568	BA8/Frontal_Mid_L (aal)	-20	22	48	4.69
	Frontal_Mid_L (aal)	-42	40	26	4.54
	Frontal_Mid_L (aal)	-28	24	48	4.12
333	BA 10/Frontal_Mid_R (aal)	38	42	22	4.68
	Frontal_Inf_Tri_R (aal)	34	30	28	4.06
	Frontal_Inf_Tri_R (aal)	42	36	10	3.87
241	Precentral_R (aal)	50	2	26	4.67

	Precentral_R (aal)	58	8	26	4.02
247	Cingulum_Mid_R (aal)	12	38	30	4.66
	Cingulum_Ant_R (aal)	4	36	28	4.2
	Cingulum_Ant_R (aal)	10	44	18	3.41
291	Paracentral_Lobule_L (aal)	-10	-16	74	4.41
	Precentral_L (aal)	-20	-18	74	4.38
	Supp_Motor_Area_L (aal)	-8	-8	58	4.07

ME, maintenance evaluation; MCE, maintenance calculation evaluation; C, competition; NC, noncompetition.

Table S6. Two-way ANOVA of the contrast values in the four interaction effect areas under competitive or non-competitive condition in the manipulation task (MCE)

Source	Type Ⅲ Sum of Squares	df	Mean Square	*F*	*P* value
**Variable: Contrast values of the right fusiform**					
Diagnosis	3.36	1	3.36	0.82	0.37
Stress	5.43	1	5.43	11.44	**0.001**
Diagnosis × Stress	6.81	1	6.81	14.37	**0.0003**
**Variable: Contrast values of the right supplementary motor area**					
Diagnosis	12.36	1	12.36	1.26	0.27
Stress	8.57	1	8.57	10.08	**0.002**
Diagnosis × Stress	13.22	1	13.22	15.55	**0.0002**
**Variable: Contrast values of the right precentral cortex**					
Diagnosis	20.25	1	20.25	3.79	0.055
Stress	2.52	2	2.52	5.45	**0.02**
Diagnosis × Stress	8.88	1	8.88	19.21	**<0.0001**
**Variable: Contrast values of the right caudate**					
Diagnosis	1.03	1		0.19	0.66

			1.03		
Stress	0.22	1	0.22	0.32	0.58
Diagnosis × Stress	9.90	1	9.90	14.42	**0.0003**

Table S7. Contrast values of the right fusiform

Right Fusiform	COMP	NON-COMP	*t*	*q* Value (FDR)
HC	0.89±1.43	0.93±1.38	0.32	0.39
OCD	1.00±1.66	0.26±1.66	4.66	**<0.0001**

COMP, competition; NON-COMP, non-competition; q Value (FDR), multiple comparisons after two-way ANOVA, FDR correction.

Table S8. Contrast values of the right supplementary motor area

Right SMA	COMP	NON-COMP	*t*	*q* Value (FDR)
HC	5.05±2.39	5.16±2.22	0.60	0.29
OCD	5.07±2.35	4.09±2.30	4.63	**<0.0001**

COMP, competition; NON-COMP, non-competition; q Value (FDR), multiple comparisons after two-way ANOVA, FDR correction.

Table S9. Contrast values of the right precentral cortex

Right Precentral	COMP	NON-COMP	*t*	*q* Value (FDR)
HC	2.00±1.67	2.21±1.80	1.60	0.06
OCD	1.78±1.58	1.09±1.72	4.37	**<0.0001**
Right Precentral	OCD	HC	*t*	*q* Value (FDR)
COMP	1.78±1.58	2.00±1.67	0.63	0.28
NON-COMP	1.09±1.72	2.21±1.80	3.11	**0.002**

COMP, competition; NON-COMP, non-competition; q Value (FDR), multiple comparisons after two-way ANOVA, FDR correction.

Table S10. Contrast values of the right caudate

Right Caudate	COMP	NON-COMP	*t*	*q* Value (FDR)
HC	1.09±1.84	1.49±1.82	2.53	**0.01**
OCD	1.41±1.37	0.87±1.82	2.83	**0.01**

COMP, competition; NON-COMP, non-competition; q Value (FDR), multiple comparisons after two-way ANOVA, FDR correction.

Table S11. Correlation between the scores on HAMA, HAMD scales and clinical symptom severity

Variables	HAMAS	HAMDS
Time (spent on or occupied with symptoms) _Obsession	**0.367***	**0.420***
Interference (with functioning or relationship) _Obsession	**0.463****	0.325
Distress (associated with the symptoms) _Obsession	**0.420***	**0.412***
YBOSS _Obsession	**0.341***	**0.343***
Interference (with functioning or relationship) _Compulsion	**0.367***	0.336
Distress (associated with the symptoms) _Compulsion	**0.372***	**0.442****

YBOSS _Obsession, the total obsession score on the Yale-Brown severity scale. * P <0.05; ** P <0.01.

Table S12. Correlation between accuracy rate, reaction time of different task patterns, and scores on clinical scales.

variables	ME_C_RT	ME_NC_RT	MCE_C_Acc	MCE_C_RT	MCE_NC_RT
HAMDTS	**-0.424****	**-0.340***	0.214	**-0.383***	**-0.336***
HAMATS	**-0.340***	-0.293	0.124	-0.299	-0.273
Time (spent on or occupied with symptom) _Compulsive	0.037	-0.046	**-0.479****	0.068	0.034
Control (of the symptom) _Compulsive	-0.100	-0.204	**-0.358***	-0.079	-0.033

ME, maintenance evaluation; MCE, maintenance calculation evaluation; Acc, accuracy; RT, average reaction time; NC, non-competition; HAMDTS, Hamilton Depression Scale total score; HAMATS, Hamilton Anxiety Scale total score. **P* <0.05; ***P* <0.01.

Table S13. Correlation between stress-related activation of the four interaction effect areas and scores on the clinical symptom severity scale in OCD patients.

OCD	R _Fusiform	R _SMA	R _Precentral	R _Caudate
Time (spent on or occupied with symptom) _Obsession	-0.244	**-0.353***	-0.247	-0.283
Interference (with functioning or relationship) _Obsession	**-0.465****	-0.314	-0.309	**-0.366***
Control (of the symptom) _Obsession	0.005	-0.069	**-0.354***	-0.202
YBOSS _Obsession	-0.211	-0.246	**-0.395***	-0.310

R_SMA, right supplementary motor area; YBOSS _Obsession, total obsession score on Yale-Brown severity scale. *, *P* <0.05, **, *P* <0.01.
